# Spatial and temporal evolution of urban economic development efficiency in China’s Yangtze River economic belt from the perspective of sustainable development

**DOI:** 10.1371/journal.pone.0273559

**Published:** 2022-09-12

**Authors:** Fengge Yao, Liqing Xue, Jiayuan Liang

**Affiliations:** School of Finance, Harbin University of Commerce, Harbin, China; China University of Mining and Technology, CHINA

## Abstract

Urban economic development is crucial to regional economy and people’s life, and enhancing the efficiency of urban economic development is of great significance to boost sustainable and healthy economic and social development. In this paper, from the perspective of sustainable development, data of 104 cities in China’s Yangtze River Economic Belt (YREB) from 2004 to 2019 are selected, and the urban resource consumption index and urban pollutant emission index are synthesized as new input-output indicators using the Time Series Global Principal Component Analysis (GPCA), combined with the Global Malmquist-Luenberger (GML) Index Model, Standard Deviation Ellipse (SDE) Model to measure the total factor productivity index of urban economic development in China’s YREB and analyze its spatial and temporal evolution. The results show that from 2004 to 2019, the total factor productivity index of urban economic development in China’s YREB showed an overall fluctuating upward trend with an average annual growth of 5.8%, and the analysis by decomposing indicators shows that the growth of total factor productivity of urban economic development in China’s YREB is mainly influenced by the growth of technological progress. Meanwhile, there are obvious regional differences in the efficiency of urban economic development in China’s YREB, with the largest difference in the middle reaches of the Yangtze River, the second largest in the upper reaches, and the smallest in the lower reaches. From 2004 to 2019, the efficiency center of gravity of urban economic development efficiency in the YREB has always been located in the middle reaches of the Yangtze River region. The spatial distribution pattern of urban economic development efficiency in the YREB is dominated by the northeast-southwest direction and tends to be concentrated in the study time period.

## Introduction

How to develop the urban economy under the premise of sustainable development is the core challenge that China urgently needs to face currently [1], and excessive rapid economic development has aggravated environmental pollution and energy waste [2]. In 2019, as an example, the percentage of 337 cities in China with excellent ambient air quality was only 31.1%, and the area of land desertification and land sanding was 2161600 square kilometers and 1721200 square kilometers, accounting for about 27% and 18% of the national land area, respectively. In accordance with the BP World Energy Statistics Yearbook released in 2017, China consumed 3.053 billion tons of oil equivalent in 2016, already surpassing the United States’ 780 million tons of oil equivalent and ranking first in the world. The increase in urban pollutants can lead to a decrease in labor supply and exacerbate urban shrinkage [3]. Meanwhile, urban pollutants are an important threat to public health [4]. And the excessive waste of energy produces a large amount of greenhouse gases and exacerbates climate disasters [5]. The YREB is a major national strategic development region in China, stretching across the eastern, central, and western parts of China, covering 11 important Chinese provinces with dense cities. As China’s economic development progresses, YERB plays a crucial role [6]. The urban clusters in the YREB contributed 44.1% of China’s GDP in 2018 [7]. However, the YREB is one of the most polluted and energy-consuming regions in China, as it gathers several key national manufacturing projects [8], and the crude production patterns of these manufacturing enterprises consume large amounts of energy and exacerbate structural pollution in the YERB. In 2014, for example, industrial emissions in the YERB reached 251432 billion cubic meters, of which NO_*x*_, SO_2_, and soot emissions were 666, 679, and 480 tons, accounting for 32%, 34%, and 28% of the corresponding national pollutant emissions, respectively [9]. Therefore, exploring a reasonable path to improve the efficiency of urban economic development in the YREB from the perspective of sustainable development, and reducing pollutant emissions as well as energy consumption while maintaining stable urban economic growth, is of non-negligible practical significance to promoting economic development and ecological civilization in the YERB.

Based on the existing researches, this paper found that the existing researches consider a single perspective on the evaluation index system of urban economic development efficiency, which needs to be further improved. Meanwhile, the existing literature lacked indicators to comprehensively measure the pollution and resource consumption levels of cities in the YERB. In addition, the existing literature on economic development efficiency from the perspective of sustainable development mainly focuses on the provincial level, and there is a less in-depth research on urban agglomerations in the YERB. Therefore, to fill these gaps, this paper focused on addressing the following two questions regarding the economic development efficiency of cities in China’s YREB from a sustainable development perspective.

- How have the temporal characteristics of the economic development efficiency of cities in China’s YREB from a sustainable development perspective changed over the past years?
- How have the spatial characteristics of urban economic development efficiency in China’s YREB changed over the past few years from a sustainable development perspective?

These questions are tightly linked to the promotion of sustainable urban development. The most important contribution of this study is to improve the evaluation framework of urban economic efficiency in terms of resource consumption, public finance, and pollutant emissions by taking 104 cities in China’s YREB as examples, and to measure urban economic development efficiency and analyze its spatial and temporal evolution characteristics from a sustainable development perspective. At the same time, this paper uses GPCA for the first time to downscale pollutant data such as industrial wastewater emissions, industrial soot emissions, industrial sulfur dioxide emissions, and resource consumption data such as total water supply, total annual electricity consumption, and total LPG gas supply, forming indicators that can be used to comprehensively measure the pollution and resource consumption levels of cities in the YREB from 2004 to 2019. This study provides a new pathway to enhance the economic development efficiency of urban clusters in the YREB from the perspective of sustainable development, which helps the government to formulate environmental protection policies, facilitate the building of ecological civilization and sustainable urban growth.

The remaining chapters of this paper are organized as follows: “literature review” section is a review of existing studies; “Materials and methods” section includes the introduction of the model, indicator selection, new indicator construction, and sample selection; “Results” section is a presentation of the empirical results; “Disscusion” section is a discussion of the empirical results, answers the two research questions raised in the introduction section, and describes the significance and limitations of this paper; and “Conclusion and Countermeasures” section shows the conclusions drawn and the policy recommendations proposed in this paper.

## Literature review

Currently, researches on the efficiency of urban economic development have focused on the following three areas.

The first is an important perspective on the efficiency of urban economic development. Several researchers have studied the factors influencing the efficiency of urban economic development. Li (2020) studied the influence of high-speed rail development on urban economic efficiency and found that the advance of high-speed rail has an important contribution to urban economic efficiency, and the longer the high-speed rail service runs, the greater the positive impact generated [10]. Zhou (2020) studied the interaction between housing prices and urban total factor productivity and found a positive and significant association between housing prices and total factor productivity [11]. Yuan (2020) used a dynamic spatial panel Durbin model and a mediating effect model to analyze the influence of manufacturing agglomeration on urban green economic efficiency, and the results showed that there is a positive “U” shaped Interaction between industrial agglomeration and urban green economic efficiency, and the upgrading of industrial structures serves as a mediator between the two [12]. Liu (2021) explored the relation and mode of transmission between technological innovation and urban green economic efficiency from the vantage point of urbanization and natural resources in 278 Chinese cities and found that technological innovation can significantly improve urban green economic efficiency [13]. Li (2021) discovered that an inverted “U-shaped” curve represented the relationship between environmental regulation and urban green economic efficiency [14]. Li (2021) used panel data from 271 Chinese cities from 2004 to 2016 to quantify urban green total factor productivity using the SBM-DEA model and examine the influence of the land market on this productivity and found that both land concession price and land concession size inhibit urban green total factor productivity growth [15]. Yu (2021) studied the effect of foreign direct investment on green total factor productivity in cities and discovered that foreign direct investment has a catalytic influence on green total factor productivity in highly clustered urban agglomerations [16]. Meanwhile, some researchers have studied the interaction between the efficiency system of urban economic development and other systems. Athanassopoulos (1997) further analyzed the relationship between economic and social efficiency by using the DEA method to measure the social and economic efficiency of Greek cities [17]. Ma (2022) studied the coordination between urban green economic efficiency and ecological welfare efficiency from the perspective of the “two mountains theory” using a coupled coordination degree model, and found that the “two mountains” theory is conducive to improving the coordinated development of green economic efficiency and ecological welfare efficiency in regional cities [18].

The second is the method of measuring the efficiency of urban economic development. According to a review of the literature, frontier analysis is the most widely used technique for assessing efficiency, which can be separated into two categories: non-parametric and parametric methods. Among the parametric methods, stochastic frontier analysis (SFA) is widely used, which measures the growth structure by using econometric methods. This method requires a predetermined production function and separates statistical errors and technical inefficiencies in the measurement process [19]. He (2009) analyzed the efficiency of 23 Chinese cities from 1978–2006 from the perspective of electricity input based on 23 typical Chinese cities selected as research samples and stochastic frontier analysis (SFA) and found that household population, fixed asset investment, urban land area, and total annual electricity consumption had positive effects on the technical efficiency of Chinese cities [20]. Dong (2020) measured the urban land use efficiency of 108 cities in the YREB using SFA based on city-level panel data from 2005–2017 and found that urban land use efficiency and industrial transformation show a synergistic effect of interactive growth in the short term, and this will have a suppressive effect on carbon emissions [21]. However, SFA also has some shortcomings. It has strict requirements for how the production function should be set and is not capable of controlling endogenous issues or input-output variables reasonably. Results may be biased if the production function is not correctly configured. [22]. Among the nonparametric methods, data envelopment analysis (DEA) is a relatively popular efficiency measure, which uses an optimization method to endogenously determine the weights of various input factors, which can avoid the specific expression of input-output relationships and can effectively compensate for the shortcomings of SFA. Xie (2015) measured the economic efficiency of urban land use under environmental constraints using a DEA model with a sequential slack-based efficiency measure based on city-level panel data from 270 cities in China. The findings indicated that most Chinese cities have much room for enhancement in the economic output of secondary and tertiary industries as well as in environmental protection efforts. The economic efficiency of land use is higher in cities with more developed economies and lower levels of pollutant emissions [23]. Zhou (2020) measured the urban green development efficiency of 285 prefecture-level cities in China using a DEA model based on urban panel data from 2005–2015. The study showed that the improvement of China’s economic strength, industrial structure, openness, and climatic conditions is conducive to improving urban green development efficiency and that the influence of social activity factors on urban green development efficiency is greater than that of natural factors [24]. Wang (2022) measured the carbon emission efficiency of the industry in China from 2002–2015 using the DEA method and analyzed its evolutionary characteristics [25]. LV (2022) selected 200 innovation indicators from 15 listed sports companies using blockchain and 35 listed sports companies not using blockchain, calculated the innovation efficiency of the sports industry using a three-stage DEA, and found that sports companies using blockchain outperformed those not using it [26]. Shah (2022) applied the DEA metafrontier to analyze the technical efficiency of 147 commercial banks in South Asia from 2013–2018, and the study found that commercial banks in Nepal had the highest technical efficiency in the region [27]. Bai (2022) applied ultra-relaxation-based measurement data envelopment analysis to measure the technical efficiency of 1219 rural Chinese hospitals in 28 provinces in China, and the study found that the hospitals performed less efficiently in terms of technology, but showed an increasing trend. In addition, there were redundancies and deficiencies in the health input and output variables, respectively [28].

The third is the selection of indicators and data to assess the efficiency of urban economic development. The evaluation system of urban economic efficiency usually consists of capital input, labor input, land input, and GDP output [29]. As the research continues to deepen, researchers have refined the evaluation system from different perspectives. Some researchers have refined the evaluation system in terms of information technology inputs. Lin (2019) accessed the economic efficiency of urban agglomerations adopting the DEA model based on the data of 20 urban agglomerations in China in 2005 and 2013 and included the number of fixed telephone users, mobile telephone users, and Internet users as input indicators in the evaluation system of urban agglomerations’ economic efficiency [30]. Some researchers have extended the evaluation framework in terms of energy inputs. Zhong (2020) analyzed the impact of energy as an input factor on the urban economy and incorporated energy inputs into the evaluation system of urban economic efficiency by selecting city-level panel data from 2008–2017 for eight cities in the YREB urban agglomeration in China and measuring urban energy economic efficiency using the SBM model. The results of the study can be seen that scale efficiency is the primary factor limiting the improvement of energy economic efficiency, and the input of production factors should be increased moderately [31]. Yang (2019) applied the game cross-efficiency DEA model to measure the urban total factor energy efficiency of 26 prefecture-level cities in China from 2005 to 2015 considering regional competitive relationships, and the results of the study showed that the urban energy efficiency considering competitive relationships was lower than the efficiency derived from traditional calculations [32]. Some researchers have updated the evaluation framework of urban economic efficiency in terms of human well-being outputs. Feng (2016) included human well-being as a socio-economic output in the efficiency evaluation system and measured and analyzed the urban sustainable development efficiency of 27 cities involving coal-fired power plants in China from 1990–2010 [33]. Wang (2019) measured and analyzed the urban green economic efficiency of 26 Chinese cities from 2005–2015 using the Super-SBM-Undesirable model based on additional consideration of human welfare factors [34]. In addition, some researchers have improved the indicator system of urban economic efficiency in terms of pollutant output. Peng (2020) selected urban sulfur dioxide emissions to represent urban pollutant emissions as a non-desired output indicator, and total urban electricity consumption to represent energy consumption as an input indicator, and applied the Malmquist index model combined with spatial autocorrelation analysis and convergence analysis to measure urban green total factor productivity in China from 2008–2016. Green total factor productivity was found to show a growing tendency during the research phase, decreasing in spatial distribution from the east to the west, and showing relatively strong spatial clustering overall [35]. Li (2021) adopted industrial sulfur dioxide emissions, industrial wastewater emissions, and industrial flue gas emissions as non-desired outputs to measure green total factor productivity in the Pearl River Delta urban agglomeration of China from 2005–2018 [36].

The above introduces important perspectives on urban economic development efficiency, measurement methods, and the selection of indicators and data. A review of the literature reveals that the studies that are currently available on the economic development efficiency of Chinese cities principally emphasize the economic efficiency of individual cities with characteristics or nationwide cities, while few studies have been conducted on the urban economic efficiency of urban agglomerations in the YREB of China. Meanwhile, the evaluation framework of urban economic development efficiency constructed from a sustainable development stance is still imperfect, and the existing assessment framework ignores the role of water resources, electricity and other resource consumption, and public finance in urban economic development. In addition, the importance of spatial heterogeneity of urban economic development efficiency is not taken into account. In terms of indicator selection, existing studies lack indicators that can comprehensively reflect the level of resource consumption and the level of various pollutant emissions in cities in the YREB. In terms of research methods, existing studies mainly focus on the analysis of the spatial evolution of urban economic efficiency in terms of spatial distribution and spatial correlation, while the analysis of the spatial center of gravity migration and pattern evolution of urban economic efficiency is rare. Therefore, this paper used 100 cities in the YREB urban agglomeration in China as samples and grouped them according to their geographical distribution, and created new indicators that can reflect urban resource consumption and various pollutant emissions comprehensively using time-series global principal component analysis. The evaluation framework of urban economic development efficiency was also established in eight aspects: capital, labor, land, resources, public finance, GDP, and pollutant emissions, and the economic development efficiency of the YREB urban agglomerations was measured. In addition, this paper used SDE for spatio-temporal evolution analysis and explored the changes in the spatial center of gravity and directional characteristics of efficiency.

The following three points primarily highlight the innovations of this paper: (1) In terms of the evaluation system, compared with the previous researches, this paper improves the evaluation framework of urban economic development efficiency, and additionally considers the influence of resource consumption such as water resources, electricity, and public finance on urban economic development efficiency, so there are innovations in the evaluation system in this paper. (2) In terms of indicators, the existing research lacks indicators that can reflect the resource consumption and pollution status of cities in the YREB. This paper uses GPCA to create a resource consumption index and pollutant emission index as new indicators. Therefore, this paper is innovative in terms of input-output indexes. In addition, the GPCA used in this paper also overcomes the shortcomings of traditional principal component analysis that cannot directly compare the scores of different years. (3) In terms of research approaches, this paper analyzes the spatial and temporal evolution characteristics and regional distribution differences of urban economic development efficiency in the YREB by combining the standard deviation ellipse model, and for the first time analyzes the spatial center of gravity, directional characteristics and changes of discrete levels of urban economic development efficiency.

## Materials and methods

### Global Malmquist¨CLuenberger (GML) index model

The Malmquist-Luenberger (ML) index can be used for dynamic efficiency measurement. It was proposed by Chung et al. (1997), which combined the directional distance function (DDF) with the traditional Malmquist index to construct a productivity index with undesired output, solving the problem that the traditional Malmquist index does not take into account undesired output [37]. According to Chung et al. the Malmquist-Luenberger Index requires the definition of a directional distance function for two different adjacent periods.

For example, suppose a production unit uses *M* inputs $x=\left(x_{1},x_{2},\ldots ,x_{M}\right)\in R_{+}^{N}$ and produces *N* desired outputs $y=\left(y_{1},y_{2},\ldots ,y_{M}\right)\in R_{+}^{N}$ and *K* undesired outputs $y=\left(y_{1},y_{2},\ldots ,y_{M}\right)\in R_{+}^{N}$. Then, its set of output possibilities in period *t* is Eq (1):

Eq (1)

$$\begin{array}{c} P^{t}\left(x^{t}\right)=\{(y^{t},b^{t})|put\;into\;x^{t}\;product(y^{t},b^{t})\},x^{t}\in R_{+}^{M},t=1,2,\ldots ,T \end{array}$$ (1)

When there is zero binding of output terms, input and desired output terms exhibit strong disposability, and undesirable outputs exhibit weak disposability, the directional distance function is introduced to increase desired outputs while reducing undesirable outputs, and its functional expression is Eq (2):

Eq (2)

$$\begin{array}{c} D^{t}(x^{t},y^{t},b^{t};g_{y},-g_{b})=max\left\{\beta \right|(y^{t}+\beta g_{y},b^{t}-\beta g_{b})\in P^{t}\left(x^{t}\right) \end{array}$$ (2)

In the above equation,*g* is the directional vector that indicates an increase in the desired output and a simultaneous decrease in the undesired output when *g* = (*g*_*y*_, −*g*_*b*_). *β* is the value of the distance function in period *t*, which indicates the maximum possible multiple of the increase in the desired output and the decrease in the undesired output.

The ML index is measured based on input-output data for each year, and the measured efficiencies are not comparable across years because the production frontier surface is different for each year. Oh (2010) proposed a Global Malmquist-Luenberger (GML) index measure for this purpose based on Chung et al.’s study [38], which requires the construction of a common production frontier using production input and output data for all periods of the sample. The current production possibility set is replaced by the global production possibility set (*P*^*G*^(*x*) = *P*^1^(*x*^1^) ∪ *P*^2^(*x*^2^) ∪ … ∪*P*^*T*^(*x*^*T*^)) to measure the gap between the technical efficiency of each period and each decision unit and the frontier in a secondary way. The functional expression of the GML index is Eq (3):

Eq (3)

$$\begin{array}{c} GML_{t+1}^{t}=\frac{1+{\vec{D}}^{G}\left(x^{t},y^{t},b^{t};y^{t},-b^{t}\right)}{1+{\vec{D}}^{G}\left(x^{t+1},y^{t+1},b^{t+1};y^{t+1},-b^{t+1}\right)} \end{array}$$ (3)

In this paper, we used the GML index to measure the change in the total factor productivity (TFP) of urban economic development, which can be decomposed into technical efficiency change (EC) and technical progress change (TC) with the following functional expression Eq (4):

Eq (4)

$$\begin{array}{c} \begin{array}{cc} GML_{t+1}^{t} & =\underset{EC_{t}^{t+1}}{\underset{︸}{\frac{1+{\vec{D}}^{t}\left(x^{t},y^{t},b^{t};y^{t},-b^{t}\right)}{1+{\vec{D}}^{t+1}\left(x^{t+1},y^{t+1},b^{t+1};y^{t+1},-b^{t+1}\right)}}}\\ & \times \underset{TC_{t}^{t+1}}{\underset{︸}{\frac{1+{\vec{D}}^{G}\left(x^{t},y^{t},b^{t};y^{t},-b^{t}\right)}{1+{\vec{D}}^{t}\left(x^{t},y^{t},b^{t};y^{t},-b^{t}\right)}\times \frac{1+{\vec{D}}^{t+1}\left(x^{t+1},y^{t+1},b^{t+1};y^{t+1},-b^{t+1}\right)}{1+{\vec{D}}^{G}\left(x^{t+1},y^{t+1},b^{t+1};y^{t+1},-b^{t+1}\right)}}} \end{array} \end{array}$$ (4)

In the Eq (4), $GML_{t}^{t+1}$ represents the change in the TFP from period *t* to *t* + 1, $EC_{t}^{t+1}$ is the change in technical efficiency from period *t* to *t* + 1, and $TC_{t}^{t+1}$ represents the technical progress from period *t* to *t* + 1. When the $GML_{t}^{t+1}$ index is greater than 1, it represents a growth in the TFP from period *t* to *t* + 1. When the $GML_{t}^{t+1}$ index is less than 1, it represents a decline in the TFP from period *t* to *t* + 1. When the $GML_{t}^{t+1}$ index is equal to 1, it represents unchanged TFP from period *t* to *t* + 1.

In this paper, following Färe et al. (1994) [39], EC is further decomposed into pure technical efficiency change (PEC) and scale efficiency change (SEC) under constant payoffs to scale (CRS), which can be expressed in a functional expression as follows Eq (5):

Eq (5)

$$\begin{array}{c} \begin{array}{cc} GML_{t+1}^{t} & =\underset{PEC_{t}^{t+1}}{\underset{︸}{\frac{1+{\vec{D}}_{VRS}^{t}\left(x^{t},y^{t},b^{t};y^{t},-b^{t}\right)}{1+{\vec{D}}_{VRS}^{t+1}\left(x^{t+1},y^{t+1},b^{t+1};y^{t+1},-b^{t+1}\right)}}}\\ & \times \underset{SEC_{t}^{t+1}}{\underset{︸}{\frac{1+{\vec{D}}_{CRS}^{G}\left(x^{t},y^{t},b^{t};y^{t},-b^{t}\right)}{1+{\vec{D}}_{VRS}^{G}\left(x^{t},y^{t},b^{t};y^{t},-b^{t}\right)}\times \frac{1+{\vec{D}}_{VRS}^{G}\left(x^{t+1},y^{t+1},b^{t+1};y^{t+1},-b^{t+1}\right)}{1+{\vec{D}}_{CRS}^{G}\left(x^{t+1},y^{t+1},b^{t+1};y^{t+1},-b^{t+1}\right)}}}\\ & \times \underset{TC_{t}^{t+1}}{\underset{︸}{\frac{1+{\vec{D}}_{VRS}^{G}\left(x^{t},y^{t},b^{t};y^{t},-b^{t}\right)}{1+{\vec{D}}_{VRS}^{t}\left(x^{t},y^{t},b^{t};y^{t},-b^{t}\right)}\times \frac{1+{\vec{D}}_{VRS}^{t+1}\left(x^{t+1},y^{t+1},b^{t+1};y^{t+1},-b^{t+1}\right)}{1+{\vec{D}}_{VRS}^{G}\left(x^{t+1},y^{t+1},b^{t+1};y^{t+1},-b^{t+1}\right)}}} \end{array} \end{array}$$ (5)

In the Eq (5), $PEC_{t}^{t+1}$ denotes the change in pure technical efficiency from period *t* to *t* + 1 and $SEC_{t}^{t+1}$ denotes the change in scale efficiency from period *t* to *t* + 1. The subscripts *CRS* and *VRS* in the equation indicate that the assumptions of constant payoffs to scale and changes in payoffs to scale are satisfied, respectively. When the decomposition index from period *t* to *t* + 1 is greater than 1, it indicates a growth in the decomposition index from period *t* to *t* + 1. When the decomposition index from period *t* to *t* + 1 is less than 1, it indicates a decrease in the decomposition index from period *t* to *t* + 1. When the decomposition index of period *t* to *t* + 1 is equal to 1, it means that the decomposition index is unchanged from period *t* to *t* + 1.

### Standard Deviation Ellipse (SDE) model

The Standard Deviation Ellipse was proposed by Lefever (1926) as a spatial statistical method that can accurately analyze the characteristics of economic spatial distribution [40]. The main parameters of the SDE include the center, the long axis, the short axis, and the azimuth. Specifically, the spatial distribution ellipse in this paper takes the mean center (MC) of the spatial distribution of the total factor productivity of urban economic development as the center and calculates its standard deviation in the X and Y directions to determine the short and long axes of the ellipse, respectively. Based on the spatial location and spatial structure of the research object, the SDE model quantitatively explains the characteristics of centrality, orientation, and spatial pattern of the total factor productivity of urban economic development in spatial allocation. The standard deviation ellipse range indicates the main area of the total factor productivity of urban economic development in spatial distribution. The mean center indicates the relative position of the distribution of the total factor productivity. The azimuth indicates the main trend direction of the total factor productivity of urban economic development in space (i.e., the angle of clockwise rotation to the long axis of the ellipse in the due north direction). The long axis indicates the dispersion of the urban economic development total factor productivity in the direction of the main trend. The functional expression of the main parameters of the SDE is as follows Eqs (6) to (9):

Eq (6)

$$\begin{array}{c} {\bar{X}}_{w}=\frac{\sum_{i=1}^{n}w_{i}x_{i}}{\sum_{i=1}^{n}w_{i}};{\bar{Y}}_{w}=\frac{\sum_{i=1}^{n}w_{i}y_{i}}{\sum_{i=1}^{n}w_{i}} \end{array}$$ (6)

Eq (7)

$$\begin{array}{c} \begin{array}{cc} \text{tan}\;\theta = & \frac{\sqrt{{\left(\sum_{i+1}^{n}w_{i}^{2}{\tilde{x}}_{i}^{2}-\sum_{i+1}^{n}w_{i}^{2}{\tilde{y}}_{i}^{2}\right)}^{2}+4\sum_{i+1}^{n}w_{i}^{2}{\tilde{x}}_{i}^{2}{\tilde{y}}_{i}^{2}}}{2\sum_{i+1}^{n}w_{i}^{2}{\tilde{x}}_{i}^{2}{\tilde{y}}_{i}^{2}}\\ & +\frac{\sum_{i+1}^{n}w_{i}^{2}{\tilde{x}}_{i}^{2}-\sum_{i+1}^{n}w_{i}^{2}{\tilde{y}}_{i}^{2}}{2\sum_{i+1}^{n}w_{i}^{2}{\tilde{x}}_{i}^{2}{\tilde{y}}_{i}^{2}} \end{array} \end{array}$$ (7)

Eq (8)

$$\begin{array}{c} \sigma_{x}=\sqrt{\frac{{\left(\sum_{i=1}^{n}w_{i}{\tilde{x}}_{i}\;\text{cos}\;\theta -\sum_{i=1}^{n}w_{i}{\tilde{x}}_{i}\;\text{sin}\;\theta \right)}^{2}}{\sum_{i=1}^{n}w_{i}^{2}}} \end{array}$$ (8)

Eq (9)

$$\begin{array}{c} \sigma_{y}=\sqrt{\frac{{\left(\sum_{i=1}^{n}w_{i}{\tilde{x}}_{i}\;\text{sin}\;\theta -\sum_{i=1}^{n}w_{i}{\tilde{x}}_{i}\;\text{cos}\;\theta \right)}^{2}}{\sum_{i=1}^{n}w_{i}^{2}}}; \end{array}$$ (9)

In Equations Eqs (6) to (9), *w*_*i*_ is the spatial location of the study object; (*x*_*i*_, *y*_*i*_) is the weight; is the weighted mean center (MC), which indicates the center of gravity of urban economic development efficiency; *θ* is the ellipse azimuth, which indicates the angle formed by the clockwise rotation to the long axis of the ellipse in the positive north direction; $\tilde{X_{i}}$ and $\tilde{X_{i}}$ indicate the coordinate deviation of each study objects location to the mean center; *σ*_*x*_ and *σ*_*y*_ are the standard deviation along the x-axis and y-axis, respectively.

### Indicator selection, data sources, and sample selection

Total factor productivity of urban economic development reflects the degree of comprehensive utilization of input factors and the proportional relationship between inputs and desired and undesired outputs in the process of urban economic development, and is often used to measure the allocation between input capital, labor, land, financial support, resources and the generated economic benefits. This paper combines the actual situation of economic development of urban agglomerations in China’s Yangtze River Economic Belt, and on the basis of previous scholars’ research, 11 indicators of 104 cities in China’s YREB urban agglomerations from 2004 to 2019 are selected as raw data following the principles of availability and representativeness of indicator selection, and the indicators are explained as follows:

1. Investment in fixed assets. A general term for the amount of work involved in the construction and acquisition of fixed assets over a certain period of time, expressed in monetary terms, and the costs associated with this [41].
2. Number of employees in urban units. Refers to the number of people working in the unit at 24:00 on the last day of the reporting period and receiving wages or other forms of labor compensation [42].
3. Built-up land area. This refers to the area of land in the urban administrative district that has actually been developed and constructed, and where municipal public facilities and public facilities are basically available [43].
4. Total water supply. Refers to the total amount of water supplied by the water supply plant outside the factory. [44]
5. Total annual electricity consumption. Refers to the sum of rural electricity consumption, industrial electricity consumption, transportation electricity consumption and urban and rural residential electricity consumption [44].
6. Total LPG gas supply. Refers to the city LPG enterprises to urban production users, household users and other users of the supply of all LPG, including outsourcing and losses [45].
7. Local general public budget expenditure. It refers to the sum of local fiscal expenditures related to general public services, public security, local social undertakings and other livelihood-related expenditures.
8. regional GDP. Refers to the final result of production activities of all resident units in a country (region) at market prices in a certain period of time [46].
9. industrial wastewater discharge. Refers to the total amount of all wastewater discharged to the outside of an industrial enterprise through all outfalls in the plant, including the production wastewater discharged outside, domestic sewage in the plant, direct cooling water and toxic and harmful mine groundwater in the mine that exceeds the discharge standards [47].
10. Industrial soot emissions. It is the sum of the total mass of soot and industrial dust emitted into the atmosphere during fuel combustion and production process of the enterprise in the reporting period [48].
11. Industrial sulfur dioxide emissions. The total amount of sulfur dioxide emitted into the atmosphere by industrial enterprises in the process of production and fuel combustion within the plant [49].

In terms of indicator processing, this paper referred to the processing method of Huang (2021) [50] and measured the capital stock data of 100 cities in the YREB during the research period by using the perpetual inventory method with 2011 as the base period based on fixed asset investment data, and deflated them. In addition, due to the lack of indicators that can comprehensively reflect the level of resource consumption and pollutant emissions of cities in the YREB in China, this paper used the GPCA method to construct new indicators, and the specific steps are as follows:

As can be seen from Table 1, the cumulative contribution of principal component 1 reaches 80.96%, which can effectively explain the consumption of the above three resources. As can be seen from Table 2, the cumulative contribution of principal component 1 and principal component 2 in the table reaches 82.05%, which can effectively explain the actual situation of pollutant emission in the above three cities.

**Table 1:** PCA results based on three types of resource consumption indicators.

Component	Eigenvalue (λ_*i*_)	Contribution Rate (%)	Cumulative Rate (%)
Comp1	2.42888	0.8096	0.8096
Comp2	0.47463	0.1582	0.9678
Comp3	0.0964905	0.0322	1

**Table 2:** PCA results based on three types of pollutant emission indicators.

Component	Eigenvalue (λ_*i*_)	Contribution Rate (%)	Cumulative Rate (%)
Comp1	1.61378	0.5379	0.5379
Comp2	0.84782	0.2826	0.8205
Comp3	0.538405	0.1795	1

Tables 3 and 4 show the eigenvectors of the principal components of resource consumption indicators and pollutant emission indicators, respectively, and the scores of the principal components can be calculated based on the eigenvectors. For resource consumption indicators, principal component 1 is selected, and the score of principal component 1 is obtained by multiplying the indicators with the corresponding eigenvectors, as shown in Eq (10).

Eq (10)

$$\begin{array}{c} f_{i}=0.613x_{1}+0.5936x_{2}+0.5215x_{3} \end{array}$$ (10)

**Table 3:** Eigenvectors of the 3 principal resource consumption components.

Variable	Comp1	Comp2	Comp3
Total water supply(*x*_1_)	0.613	-0.2676	-0.7434
Total annual electricity consumption(*x*_2_)	0.5936	-0.465	0.6568
Total LPG gas supply(*x*_3_)	0.5215	0.8439	0.1262

**Table 4:** Eigenvectors of the 3 principal pollutant emission components.

Variable	Comp1	Comp2	Comp3
Industrial wastewater discharge(*y*_1_)	0.6316	-0.3248	0.704
Industrial fume emissions(*y*_2_)	0.4458	0.895	0.013
Industrial SO2 emissions(*y*_3_)	0.6343	-0.3057	-0.7101

For the pollutant emission category indicators, principal component 1 and principal component 2 were selected, and the scores of principal component 1 and principal component 2 were obtained by multiplying the indicators with the corresponding feature vectors, as shown in Eqs (11) and (12) respectively.

Eq (11)

$$\begin{array}{c} c_{1}=0.6316y_{1}+0.4458y_{2}+0.6343y_{3} \end{array}$$ (11)

Eq (12)

$$\begin{array}{c} c_{2}=-0.3248y_{1}+0.895y_{2}-0.3057y_{3} \end{array}$$ (12)

The calculated principal component scores were multiplied by the corresponding variance contributions and then summed up and divided by the cumulative contribution. The total principal component scores of resource consumption indicators and urban pollutant emission indicators can be obtained separately. The calculation formulas are as Eq (13):

Eq (13)

$$\begin{array}{c} F=f_{1};C=(0.5379c_{1}+0.2826c_{2})/0.8205 \end{array}$$ (13)

Finally, the total principal component scores of resource consumption indicators and the total principal component scores of urban pollutant emission indicators were normalized to obtain the urban resource consumption index and urban pollutant emission index, respectively.

In terms of the index system, this paper establishes the input-output index system from seven aspects: capital input, labor input, land input, resource input, financial input, economic output, and pollutant output. Among them, capital stock is used as capital input index, the number of urban employees per unit is used as labor input index, the land area of built-up area is used as land input index, urban resource consumption index is used as resource input index, local general public budget expenditure is used as public financial input index, regional GDP is used as economic output index, and urban pollutant emission index is used as pollutant output index. The data in this paper were obtained from the China Urban Statistical Yearbook, and some missing data have been smoothed. The descriptive statistics of the indicators are shown in Table 5.

**Table 5:** Descriptive statistics of input and output indexes.

Criterion lLayer	Index lLayer	Unit	Max.	Min.	Mean	Std.Dev.
Input indexes	Capital stock	10^9^ yuan	8.47*10^4^	2.47*10^2^	5.93*10^3^	7.92*10^3^
	Number of employees in urban units	10^4^ people	9.87*10^2^	5.5	57.2	88.39
	Built-up land area	km2	1.5*10^3^	10	1.24*10^2^	1.67*10^2^
	Urban resource consumption index		1.00	0.00	0.06	0.11
	Local general public budget expenditure	10^4^ yuan	8.35*10^7^	7.91*10^4^	3.04*10^6^	5.73*10^6^
Desired output indicators	Regional GDP	10^4^ yuan	3.82*10^8^	5.04*10^5^	2.1*10^7^	3.29*10^7^
Undesired output indicators	Urban pollutant emission index		1.00	0.00	0.04	0.05

In terms of sample selection, China’s YREB includes 11 provinces and cities in China, spanning three regions in the east, middle and west of China by the Yangtze River waterway, covering 21.4% of China’s land area, gathering 42.8% of China’s population, with dense urban distribution, and creating 44.1% of China’s GDP in 2018. Currently, China’s economic development has shifted from the high-speed growth stage to the high-quality development stage, and the YREB has been strategically defined by the Central Committee of the Communist Party of China and the State Council as the main force leading China’s high-quality economic development due to its unique transportation advantages, abundant resources, superior industries, and dense human resources. In this paper, 104 cities in China’s YREB are selected as samples, and according to the high, middle, and lower sections of the Yangtze River, 104 cities in China’s YREB are split into these three zones, as shown in Table 6.

**Table 6:** Samples selected from 2004 to 2019.

Region	Province	Cities
Upper Yangtze	Sichuan	Bazhong, Chengdu, Dazhou, Deyang, Guangyuan, Leshan, Luzhou, Meishan, Mianyang, Nanchong, Neijiang, Panzhihua, Ya’an, Yibin, Ziyang, Zigong
	Yunnan	Baoshan, Kunming, Lijiang, Lincang, Qujing, Yuxi, Zhaotong
	Guizhou	Anshun, Guiyang, Liupanshui, Zunyi
		Chongqing (municipality)
Middle Yangtze	Hubei	Ezhou, Huanggang, Huangshi, Jingmen, Jingzhou, Shiyan, Suizhou, Wuhan, Xianning, Xiaogan, Yichang
	Hunan	Changde, Chenzhou, Hengyang, Huaihua, Loudi, Shaoyang, Xiangtan, Yiyang, Yongzhou, Yueyang, Zhangjiajie, Changsha, Zhuzhou
	Jiangxi	Fuzhou, Ganzhou, Ji’an, Jingdezhen, Jiujiang, Nanchang, Pingxiang, Shangrao, Xinyu, Yichun, Yingtan
	Anhui	Anqing, Bengbu, Bozhou, Chizhou, Chuzhou, Fuyang, Hefei, Huaibei, Huainan, Huangshan, Liuan, Maanshan, Tongling, Wuhu, Suzhou (city in Anhui), Xuancheng
Lower Yangtze	Jiangsu	Changzhou, Huaian, Lianyungang, Nanjing, Nantong, Suzhou (city in Jiangsu), Taizhou (city in Jiangsu), Wuxi, Suqian, Xuzhou, Yangzhou, Zhenjiang
	Zhejiang	Hangzhou, Huzhou, Jiaxing, Jinhua, Lishui, Ningbo, Quzhou, Shaoxing, Taizhou (city in Zhejiang), Wenzhou, Zhoushan
		Shanghai (municipality)

## Results

### Temporal evolution of the TFP for urban economic development

**Fig 1:**
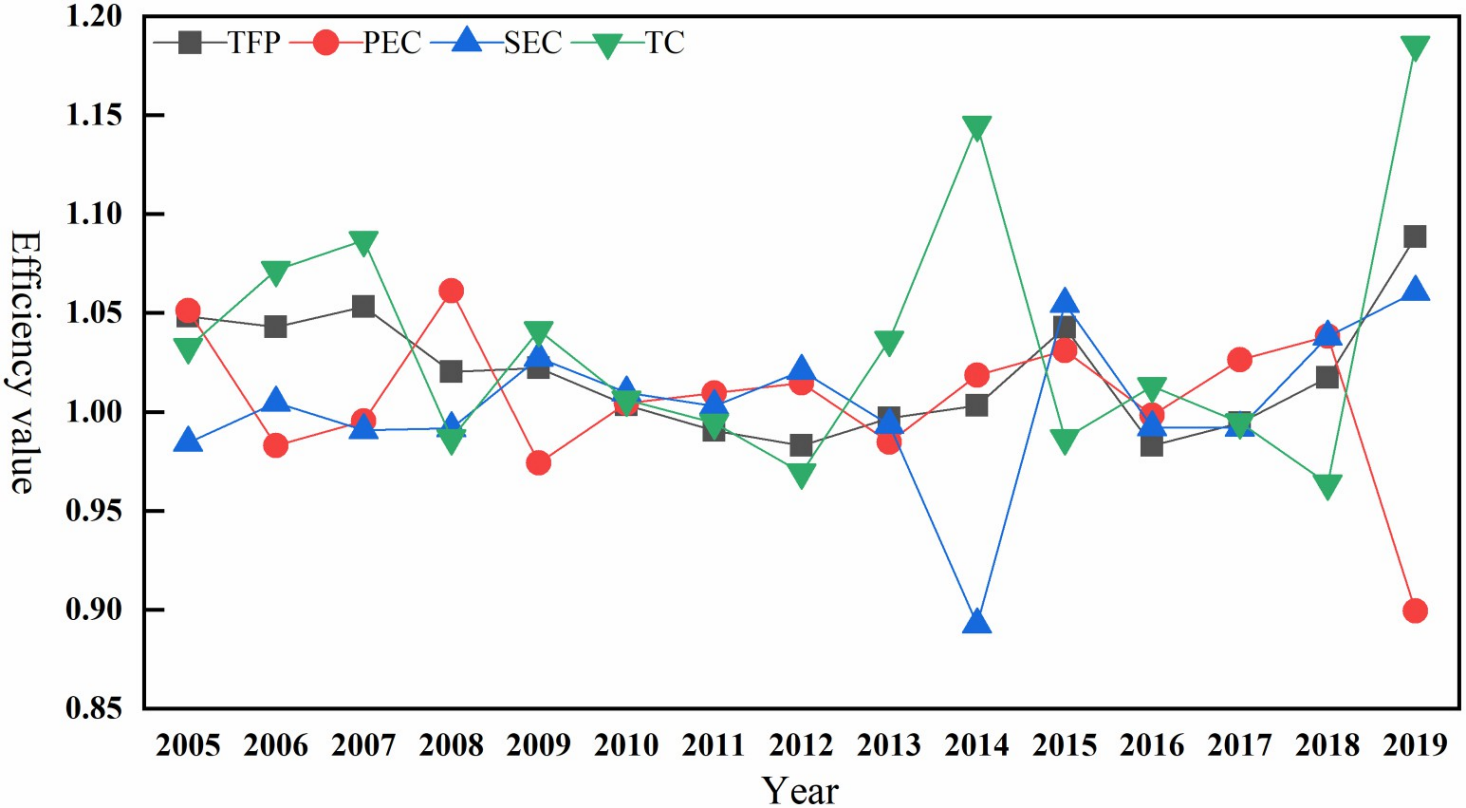
Changes in the numerical distribution of TFP for urban economic development in 2004–2019.

Fig 1 showed the distribution of the values of TFP for urban economic development in China’s YREB from 2004 to 2019 and the changes in the degree of dispersion, with the error bars in the figure corresponding to 1.5 times the standard deviation of TFP and the yellow pentagrams corresponding to the outliers of TFP. From an overall perspective, the TFP of urban economic development in China’s YREB is at a relatively high level during the 15 years from 2004 to 2019. The years of TFP growth have amounted to 10 years, accounting for 66.7% of the whole 15 years, and although there were 5 years with negative TFP growth, the negative growth rate was low. In addition, the variance level of TFP values of urban economic development in China’s YREB from 2004 to 2019 showed an overall trend of first decreasing and then increasing. In terms of specific periods, the difference between the maximum and minimum values of urban economic development efficiency gradually decreased from 0.958 in 2004–2005 to 0.282 in 2009–2010, and the different levels of urban economic development efficiency in the YREB reached its lowest level in 2009–2010. After 2010, the difference in urban economic development efficiency showed fluctuating growth from 0.382 in 2011–2012 to 0.835 in 2018–2019, and the different levels of urban economic development efficiency reached their peak in 2014–2015 with an efficiency difference of 1.377.

**Fig 2:**
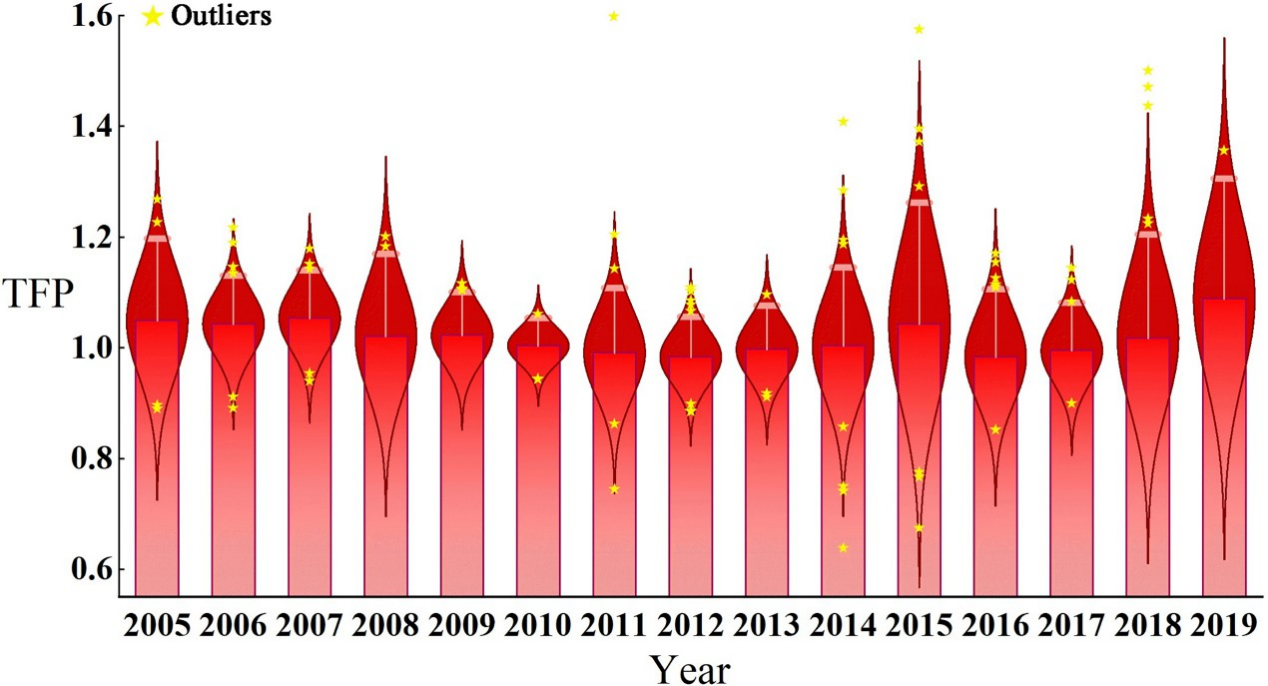
Changes in TFP for urban economic development and changes in decomposition indicators in 2004–2019.

From a temporal perspective, as shown in Fig 2, the TFP for urban economic development in the YREB of China showed an overall fluctuating and gradual upward trend from 2004–2019, with an average yearly rise of 5.8%. 2018–2019 showed the largest rise with an increase of 8.5%, and 2011–2012 and 2015–2016 showed the largest decline, both by 1.7%. Urban economic development TFP increased by 4.8%, 4.3%, 5.3%, 2%, 2.2%, and 0.4% per year between 2004–2010, respectively. Urban economic development TFP declined by 0.09%, 1.7% and 0.3% per year between 2010 and 2013, respectively. This indicated that the long-established traditional industries characterized by high pollution, high energy consumption, and high emissions have brought a series of adverse chain reactions to urban economic development, including resource constraints, environmental pollution, climate change, and other urban problems that human beings cannot avoid, and the sustainability of urban economic development has suffered from pressure. From 2013–2014, urban economic development TFP showed an increase with a growth rate of only 0.3%, and decomposition of urban economic development TFP can be known that technological progress grew by 14.5%, PEC grew by 1.9%, and SEC decreased by 10.7%. This showed that the TFP growth was primarily due to the growth of TC, while the lower growth rate was due to a significant decrease in scale efficiency. From 2014 to 2019, urban economic development TFP maintained growth with growth rates of 4.3%, 1.7%, and 8.9% in 2014–2015, 2017–2018, and 2018–2019, except for a small decrease in TFP of 1.7% and 0.5% in each year from 2015–2017, where 2014–2015, and TFP growth was higher in 2018–2019. This showed that within these seven years the government enacted sustainable economic development policies advocating energy conservation and emission reduction, clean energy development, improved energy utilization efficiency, further optimization of energy industry structure, substantial technological progress, effective control of industrial emissions, and significant improvement of economic efficiency and market competitiveness.

In terms of decomposition indicators, the trends of PEC, SEC, and TC from 2004 to 2019 were basically consistent with those of TFP, with an average annual growth of 0.6% in PEC, only 0.3% in SEC and 4.8% in TC, indicating that the main reason for the rise in the TFP index was the growth of TC. At the same time, SEC only increased by 0.3%, indicating that the urban economic growth dividend brought by SEC was diminishing. In terms of specific time periods, the changes in the decomposition index are larger in 2013–2014, with TC increasing by 14.5%, PEC increasing by 1.9%, and SEC decreasing by 10.7%, which shows that TFP only increased by 0.3% in 2013–2014, mainly driven by the growth of TC, while SEC limited the growth of TFP. In addition, the decomposition indicators also produced substantial changes in 2018–2019, with TC showing the most substantial growth, with a growth rate of 18.6%. Meanwhile, SEC also maintained its growth, with a growth rate of 6%. Only PEC decreased by 0.1%. Analysis of the decomposition indicators showed that the main reasons for the growth of TFP in urban economic development in 2018–2019 were the growth of TC and SEC, with a greater contribution of TC.

### Spatial evolution of the efficiency of urban economic development

**Fig 3:**
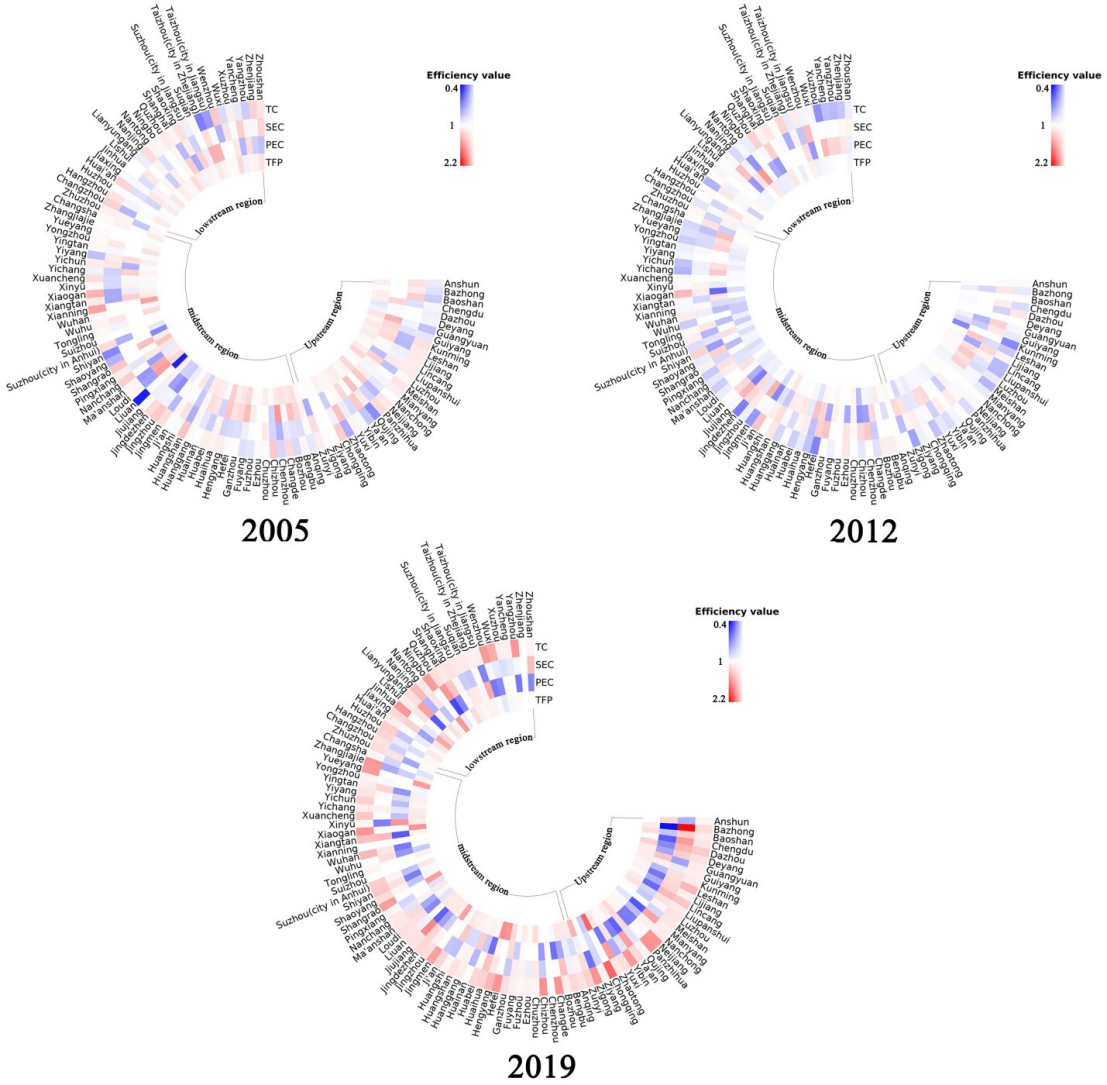
Spatial distribution of urban economic development TFP indicators and decomposition indicators.

From the spatial perspective, there were obvious regional differences in TFP of urban economic development in China’s YREB, with the largest difference in the middle Yangtze River region, followed by the upstream region, and the smallest difference in the downstream region, as shown in Fig 3. In this paper, 2005, 2012, and 2019 were selected as time points to analyze the TFP of urban economic development as well as the decomposition indexes. In 2005, the overall level of TFP of urban economic development in the YREB was relatively good, with 84 cities with TFP growth, accounting for 80.8% of the selected sample size. Among them, Xianning, Shangrao, and Hengyang had TFP growth of more than 20%, which were concentrated in the middle and lower reaches of the Yangtze River. 20 cities did not experience TFP growth, which was mainly distributed in the middle and lower reaches of the Yangtze River. Among them, the TFP of Liu’an and Shiyan showed a large decline. Compared with 2005, some cities with original TFP growth turned into negative TFP growth in 2012, mainly concentrated in the upper and middle reaches of the Yangtze River region. The number of cities with TFP growth decreased from 84 to 35, accounting for only 33.7% of the sample, with 8 cities distributed in the upper Yangtze River region, and 16 cities distributed in the middle Yangtze River region, and 11 cities distributed in the lower Yangtze River region. Among the 104 selected sample cities, there are no cities with the TFP growth of more than 20%, and only one city, Kunming, has negative TFP growth of more than 20%. In 2019, the majority of cities in China’s YREB experienced growth in TFP change in economic development, with 85 cities, accounting for 81.7% of the selected sample, of which the number of cities with TFP growth exceeding 20% was 11, primarily located in the upstream and midstream regions of the Yangtze River basin, namely Chongqing, Hengyang, Xiaogan, Hefei, Ningbo, Yueyang, Shangrao, Jingmen, Jinhua, Zunyi, and Dazhou.

In terms of decomposition indicators, in 2005, the growth of urban economic development efficiency in Xianning, Shangrao, and Hengyang, on the one hand, was outstanding. Among them, the PEC and SEC of Xianning and Shangrao were both 1, while the TC increased by 42.8% and 26.7% respectively, thus we can know that the TFP growth of Xianning and Shangrao was driven by the growth of technical progress. Hengyang’s SEC showed negative growth in 2005, declining by 9.8%. Hengyang’s TC and PEC both showed growth in 2005, up by 7.7% and 26.3% respectively, so TC and PEC growth were the main reasons for Hengyang’s TFP growth, with the most significant role of PEC. On the other hand, the urban economic development efficiency of Lu’an and Shiyan showed a large decline, and the PEC and SEC did not show any change, while the TC declined significantly, by 52.9% and 27.6%, respectively, so the decline of TFP in these two cities was negatively affected by the decline of TC. In 2012, there was no city with outstanding growth in urban economic development efficiency, and Kunming was the only city with urban economic development efficiency declining by more than 20%. The city’s PEC and SEC showed no change, and only TC grew by 27.4%, thus showing that Kunming’s TFP was mainly driven by TC. In 2019, Chongqing, Hengyang, Xiaogan, Hefei, Ningbo, Yueyang, Shangrao, Jingmen, Jinhua, Zunyi, and Dazhou had rapid growth in urban economic development efficiency, and all of the above 11 cities had a substantial increase in TC of more than 20%. Except for Hengyang, which showed a small increase of 3.7% in PEC, the remaining 10 cities did not show an increase in PEC. While the SEC of Hengyang, Zunyi, and Dazhou changed to growth, with Hengyang and Zunyi increasing by 10.9% and 14.5% and Dazhou significantly increasing by 33.5%, the SEC of the remaining eight cities did not show growth. It can be seen that the TFP growth in Chongqing, Hengyang, Xiaogan, Hefei, Ningbo, Yueyang, Shangrao, Jingmen, Jinhua, and Zunyi was mainly influenced by the growth of TC, and the TFP growth in Dazhou was mainly influenced by the combination of TC and SEC.

**Fig 4:**
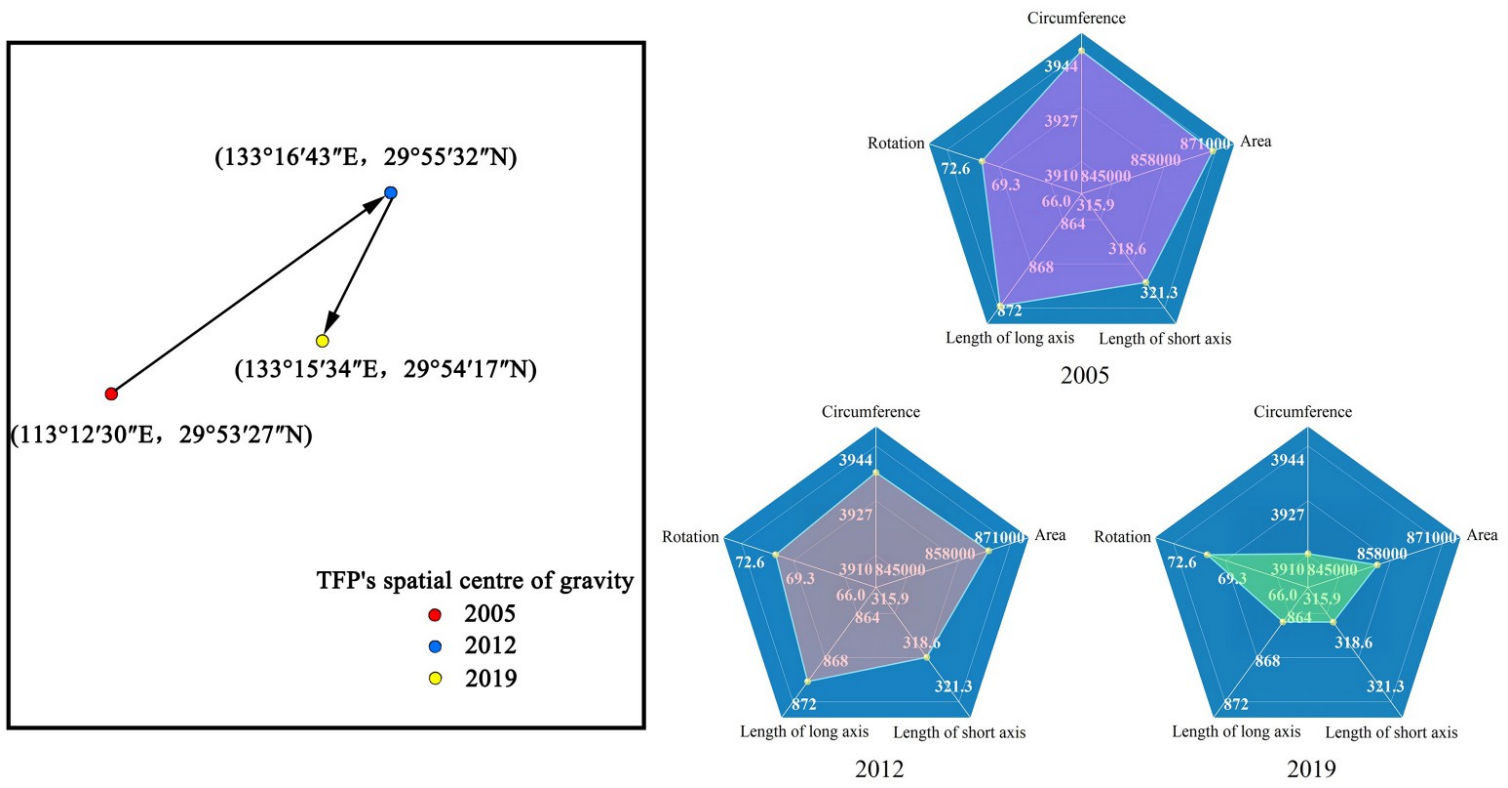
Spatial center of gravity of TFP and the parameter variation of SDE.

It is important to analyze the migration of the center of gravity of efficiency and the change of SDE to analyze the spatial evolution of urban economic development TFP. The center of gravity of efficiency can reflect the spatial distribution of urban economic development TFP in the YREB, and SDE can reflect the spatial dispersion of urban economic development TFP in the YREB. Therefore, this paper adopted the SDE model to analyze the TFP of urban economic development. In this paper, based on the urban economic development efficiency in 2005, 2012, and 2019, the relevant parameters of SDE were obtained using Arcgis 10.2 software, and the spatial center of gravity migration trajectory of TFP and the parameter changes of SDE were shown in Fig 4.

In terms of the spatial center of gravity migration of TFP, the center of gravity of urban economic development TFP in 2005–2019 is in the range of (E113°12′30″–E113°16′43″,N29°53′27″–N29°56′32″) and always located in the middle reaches of the YREB. This indicates that the urban economic development TFP of cities in the YREB is relatively stable in space during the study period, showing the spatial distribution of strong midstream and weak upper and lower reaches. From different periods, the center of gravity of urban economic development TFP shifted to the northeast from 2005 to 2012, and the center of gravity of urban economic development TFP shifted to the southwest from 2012 to 2019. Analyzing the reasons for the above changes, we can see that the TFP of cities located in the northeast, such as Taizhou (city in Jiangsu), Nantong, and Anqing, generally showed an increasing trend from 2005 to 2012, and the TFP of cities in the southwest of the YREB generally displayed a significant downward trend from 2005 to 2012, so the center of gravity moved to the northeast. In contrast, the TFP of cities in the southwestern part of the YREB generally changed from a downward trend to an upward trend after 2012, and the TFP showed a significant increase, thus forming a trajectory of the center of gravity to the southwest.

In terms of changes in SDE, the range of changes in the long axis of the ellipse from 2005 to 2019 is from 864 to 871 km, the range of changes in the short axis is from 316 to 319 km, and the range of the rotation angle of the SDE is from 71.51° to 71.60°. The spatial distribution pattern of TFP for urban economic development in the YREB is stable, showing a spatial distribution trend dominated by the northeast-southwest direction. Further analysis by periods shows that the range of SDE distribution from 2005 to 2012 shows a trend of narrowing, the long and short axes are shortened, and the ellipse rotation angle is unchanged, which means that the spatial distribution of TFP of urban economic development in the YREB tends to be concentrated, and the dispersion of TFP distribution in the direction of long and short axes has weakened. The range of the standard deviation ellipse distribution from 2013 to 2019 shows a further narrowing trend, with both the long and short axes shortening and the ellipse rotation angle remaining unchanged, indicating that the spatial distribution of TFP for urban economic development in the YREB continues to maintain a concentrated trend, and the imbalance in the direction of the long and short axes continues to weaken.

## Disscusion

This paper evaluated the resource consumption index and pollutant emission index of 104 cities in China’s YREB, and also measured the urban economic development efficiency from the viewpoint of sustainable development, and analyzed the spatial and temporal evolutionary trends of urban economic development efficiency and the changes in the spatial center of gravity and directional characteristics. These findings can help policy research departments to understand the path of improving the economic development efficiency of urban agglomerations in China’s YREB, to better promote urban economic development and ecological civilization in the region and improve the level of sustainable urban development.

First, in terms of temporal evolution, the economic development efficiency of cities in China’s YREB was generally at a relatively high level during the 15 years from 2004 to 2019, with only a small decline in individual years. This is because, starting from 2004, the Chinese government has improved the quality of employment through a series of policies to enhance sustainable development, which effectively promotes domestic consumption and pulls the cities’ stable economic growth. Meanwhile, the cities in the YREB have strictly implemented energy conservation and emission reduction policies to improve energy utilization and effectively control industrial pollutant emissions, promoting the transformation of the urban economic development model to an intensive economic development mode. As a result, the TFP of urban economic development in the YREB in China is generally high. However, as some local governments try to protect the local economy and set up various policy barriers, the products and factors cannot realize the free flow across regions and sectors without obstacles, so the labor market and capital market between different regions and sectors have misplaced resources. As a result, the TFP of urban economic development in China’s YREB showed a negative growth rate with a low growth rate in a few years. For this reason, the Chinese government should formulate relevant policies to boost the transformation and upgrading of local industries, improve the core competitiveness of local enterprises, eliminate policy barriers, and promote the free flow of products and factors. In terms of decomposition indicators, the growth of economic development efficiency of cities in China’s YREB from 2004–2019 was mainly driven by TC. The high growth of TC means that the economic development of cities in the YREB made important progress in technological innovation from 2004 to 2019. On the one hand, advances in information and communication technology accelerated the development of e-commerce, which greatly improved market efficiency and optimized resource allocation for urban economic growth through interest leverage. The development of e-commerce also promoted the improvement and upgrading of industrial structure and reduced the proportion of traditional industries in the economy, thus reducing resource consumption, mitigating the damage of traditional industries to resources and the environment, and promoting the sustainable development of the urban economy. On the other hand, technological progress in energy development and utilization has led to a significant increase in energy utilization and effective control of pollutant emissions. In terms of specific periods, the TFP growth in 2014–2015 was significant, growing by 4.3%, which was second only to the TFP growth in 2018–2019. This may be because China’s State Council introduced policies to strengthen air pollution control, such as haze, in 2014, encouraging enterprises to adopt advanced technologies to proactively treat pollution and reduce emissions, forcing highly polluting enterprises to exit, ensuring that the urban economy achieves sustainable development, resulting in faster growth of urban economic development TFP. This may be because China’s State Council introduced policies to strengthen air pollution control, such as haze, in 2014, encouraging enterprises to adopt advanced technologies to proactively treat pollution and reduce emissions, forcing highly polluting enterprises to exit, ensuring that the urban economy achieves sustainable development, resulting in faster growth of urban economic development TFP. The growth rate of urban economic development TFP in 2018–2019 was the largest in 15 years, with a growth rate of 8.9%. This may be due to the three-year extension of the purchase tax exemption for new energy vehicles in 2018, which greatly stimulated the research and development efforts of the new energy industry, new energy technologies got a stage breakthrough, urban pollutant emissions were further controlled, and urban economic sustainability made greater progress, thus urban economic development TFP had greater growth in 2018–2019. In summary, this paper here discussed the time evolution characteristics of urban economic development efficiency in China’s YREB from 2004–2019 from the perspective of sustainable development, answering the first question raised in “Introduction” section.

Second, in terms of spatial evolution, the efficiency of urban economic development in China’s YREB had regional differences, mainly showing a distribution with the largest differences in the middle reaches of the Yangtze River, followed by the upstream regions and the smallest differences in the downstream regions. This is mainly because the cities in the middle reaches of the Yangtze River are at different levels and stages of development, with large differences in economic development levels and loose economic ties, and the capital cities of Wuhan, Changsha, Nanchang and Hefei have a “siphon effect” on the resources of the surrounding areas. At the same time, the market in the midstream region is severely fragmented, and the financial market, land factor market, environmental technology and information market, innovation factor market, and other factor markets have not yet formed unified access standards and technical standards, which is not conducive to the realization of inter-city synergistic development, so the differentiation of TFP in the region is most obvious. The cities in the downstream region are relatively close to each other in terms of development level, and the cities with high economic development levels have strong radiation levels, and the “siphon effect” of the provincial capital cities on the resources in the surrounding areas is weak. Compared with the above two regions, the geographical, economic and resource conditions of cities in the upstream region are in between, so the level of differentiation among cities in this region is between the midstream and downstream regions. From the change of the spatial center of gravity of TFP, the center of gravity of urban economic development TFP from 2005 to 2019 was consistently located in the midstream region of the YREB moving within the midstream region. This is mainly due to the superior geographical conditions of the midstream region, which connects the upstream and downstream regions. With the construction and connection of infrastructure in the midstream region, the transportation network has been improved, and the exchange of resources in science and technology, industry, and talents tends to be frequent, which accelerates the advance of the urban economy in the region. Meanwhile, the urban clusters in the middle reaches of the Yangtze River region are developing rapidly in manufacturing and urbanization, which is conducive to further expanding investment and boosting consumption to promote economic growth. In addition, the region is one of the most densely populated regions in China in terms of freshwater resources, with outstanding environmental carrying capacity, and with industrial transformation and upgrading, the level of sustainable development in the region has been rising. Therefore the center of gravity of urban economic development TFP always moved in the midstream region. In summary, this paper here discussed the spatial evolutionary characteristics of urban economic development efficiency in China’s YREB from 2004–2019 from the perspective of sustainable development, answering the second question raised in “Introduction” section.

In addition to the above answers to the research questions, this paper provided several novel contributions to relevant studies. Firstly, in terms of urban economic development efficiency evaluation, this paper complemented the framework for evaluating urban economic development efficiency in previous studies from a sustainable development perspective [29]. Compared with existing studies, this paper improved the evaluation framework of urban economic development efficiency in terms of water resources, electricity and other resource consumption, and public finance. Other studies on urban economic development efficiency can be further expanded based on this, such as the influence of environmental regulation on urban economic development efficiency [14]. Second, this study provided a new perspective for quantitative analysis by using the GPCA model to measure the resource consumption and pollutant emission levels of cities in the YREB, which helps relevant departments to understand the comprehensive changes of cities in terms of water resources, electricity resources, and industrial waste emissions. Third, this paper further analyzed the spatial and temporal evolution and spatial center of gravity migration trajectory of urban economic development efficiency, which will be helpful to explore the path of improving urban economic development efficiency and provide some basis for local governments to formulate policies to promote sustainable urban development.

In addition, there are still some limitations in this paper. First, this work used the most recent data that is currently obtainable, but there is still some lag, for further research can be done after the data is updated. Secondly, this paper did not take into account climate and people’s welfare in the framework of urban economic development efficiency evaluation, which can be supplemented in future studies. Third, this paper failed to explore the factors influencing the efficiency of urban economic development and the future trend changes, which can be further expanded in future research.

## Conclusion and countermeasures

### Conclusion

This paper synthesizes the new input and output indicators using the GPCA model, and calculates the TFP and its decomposition index of urban economic development in China’s YREB from 2004 to 2019 using the GML Model. In addition, this paper analyzes the time evolution of urban economic development efficiency of 104 cities in China’s YREB and the spatial evolution of urban economic development efficiency using SDE Model, which provides an effective theoretical basis for improving the urban economic development efficiency of China’s YREB. The conclusions are as follows:

1. Summarizing from the perspective of temporal evolution, it can be seen that the overall TFP of urban economic development in China’s YREB exhibited a fluctuating upward tendency from 2004 to 2019, with an average annual growth of 7.3%. At the same time, PEC grew 0.6%, SEC grew 0.3%, TC grew 4.8%, and the rise of TFP was mainly driven by TC.
2. Summarizing from the perspective of spatial differences, it can be seen that there were obvious regional differences in the overall TFP of urban economic development in China’s YREB from 2004 to 2019, with the largest differences in the middle reaches of the Yangtze River, the second largest in the upper reaches, and the smallest in the lower reaches. For specific cities, the TFP growth of Xianning, Shangrao, and Hengyang exceeded 20% in 2005, no cities with TFP growth of more than 20% in 2012, and the TFP growth of Chongqing, Hengyang, Xiaogan, Hefei, Ningbo, Yueyang, Shangrao, Jingmen, Jinhua, Zunyi, and Dazhou exceeded 20% in 2019.
3. Summarizing from the perspective of spatial center of gravity migration and spatial pattern changes, it can be seen that the center of gravity of efficiency of urban economic development efficiency in the YREB from 2004 to 2019 was always in the middle reaches of the Yangtze River region, showing a trajectory of moving first to the northeast and then to the southwest. The efficiency of urban economic development showed spatially unbalanced development, and typically demonstrated a tendency of moving up from the middle Yangtze River region and down from the downstream region, and the efficiency of urban economic development in the YREB showed a spatial distribution trend dominated by the northeast-southwest direction, and there was a concentration trend in spatial distribution.

### Countermeasures and suggestions

Based on the results of the above analysis, in order to better improve the efficiency of urban economic development, this paper puts forward the following three policy recommendations:

1. Increase policy support for environmental technology innovation to expand the advantages in terms of TC. The current growth in economic development efficiency of cities in the YREB is mainly driven by technological progress, therefore, support for technological innovation and introduction should be increased at the policy level to further expand the advantages of sustainable urban economic development in the region. We should enhance investment in scientific research, rise the cities’ technological innovation capabilities in energy use and environmental protection, attract domestic and foreign experts in energy and environmental protection, train professional scientific researchers, conduct regular talent exchange activities, and build a quadrilateral platform centered on science and technology industrial parks, universities, scientific research institutions, and local governments to maximize knowledge spillover effects and boost sustainable urban economic development. In addition, according to the slow growth of SEC in the region, the government can also design different performance appraisal standards for enterprises of different nature, improve financial and tax incentive policies, and set up energy tax, environmental tax, and other taxes to actively boost the management and promotion level of energy-saving and emission reduction technologies of enterprises.
2. Improve the level of coordinated development of economic development efficiency among cities. There were spatial differences in the economic development efficiency of cities in the YREB, and the development was unbalanced. For the cities in the midstream region, they should strengthen the exchange of technology and talents, so that the cities with higher urban economic development efficiency can drive the rapid growth of the cities with lower efficiency, thus improving the coordination level of the region. And the cities in the upstream and downstream regions should strengthen the flow of production factors between cities, make full use of each city’s own advantages, break the regional barriers of differentiated industries, strengthen the rationalization of resource allocation in neighboring areas, and further enhance the sustainability and coordination of urban economic development.
3. Increase the investment and construction in the southwestern part of the YREB. According to the trajectory that the spatial center of gravity of urban economic development efficiency shifted first to the northeast and then to the southwest during the study period, there is room for higher development of urban economic development efficiency in the southwestern region. The natural conditions of cities in the southwest should be fully utilized to develop the local tourism economy, promote industrial structure upgrading, pull local employment increase, and maintain a high level of economic development efficiency while ensuring low pollution. In addition, some cities with superior resource conditions can reasonably develop clean energy such as hydroelectricity and wind power generation according to the actual situation to promote sustainable urban economic development.

## Supporting information

S1 Table TFP and decomposition index of urban economic development from 2004 to 2019. (DOCX)
