# Supplementary material for: Spatial and temporal evolution of urban economic development efficiency in China’s Yangtze River economic belt from the perspective of sustainable development
Source: PLoS One. 2022 Sep 12;17(9):e0273559. doi: 10.1371/journal.pone.0273559 (PMC9467368; doi:10.1371/journal.pone.0273559)
Supplement: S1 Table — (DOCX) [file pone.0273559.s001.docx]

**S1 Table. TFP and decomposition index of urban economic development from 2004 to 2019.**

| YEAR | TFP | TC | SEC | PEC |
| --- | --- | --- | --- | --- |
| 2004/2005 | 1.048 | 1.033 | 0.984 | 1.051 |
| 2005/2006 | 1.043 | 1.072 | 1.005 | 0.983 |
| 2006/2007 | 1.053 | 1.087 | 0.991 | 0.996 |
| 2007/2008 | 1.020 | 0.987 | 0.992 | 1.061 |
| 2008/2009 | 1.022 | 1.041 | 1.027 | 0.974 |
| 2009/2010 | 1.004 | 1.006 | 1.010 | 1.004 |
| 2010/2011 | 0.991 | 0.995 | 1.003 | 1.010 |
| 2011/2012 | 0.983 | 0.969 | 1.021 | 1.015 |
| 2012/2013 | 0.997 | 1.037 | 0.994 | 0.985 |
| 2013/2014 | 1.003 | 1.145 | 0.893 | 1.019 |
| 2014/2015 | 1.043 | 0.987 | 1.055 | 1.031 |
| 2015/2016 | 0.983 | 1.013 | 0.992 | 0.999 |
| 2016/2017 | 0.995 | 0.995 | 0.992 | 1.026 |
| 2017/2018 | 1.017 | 0.964 | 1.038 | 1.038 |
| 2018/2019 | 1.089 | 1.186 | 1.061 | 0.899 |
| MEAN | 1.019 | 1.034 | 1.004 | 1.006 |
